# Third molars surgical extraction awareness: a cross-sectional questionnaire-based survey

**DOI:** 10.1007/s10006-026-01572-2

**Published:** 2026-05-27

**Authors:** Jiří Borovec, Wanda Urbanová, Petra Křížová, Jana Vašáková, David Vařejčko, Petra Poláčková

**Affiliations:** 1 Department of Stomatology, 3rd Faculty of Medicine, Charles University and University Hospital Královské Vinohrady, Šrobárova 50, Prague 10, 10034 Czech Republic; 2 3rd Faculty of Medicine, Charles University, Prague, Czech Republic; 3 Prague Cleft Center, University Hospital Královské Vinohrady, Prague, Czech Republic; 4 Faculty of Medicine in Pilsen, Charles University, Pilsen, Czech Republic

**Keywords:** Third molar, Surgical extraction, Public awareness

## Abstract

**Purpose:**

This study aimed to assess public awareness of the surgical third molar extraction, preventive recommendations, and fear levels among individuals who underwent third molar surgical extraction (E) and those without prior experience (WE).

**Methods:**

A questionnaire survey of 584 respondents assessed demographics, knowledge of preventive recommendations, areas in which they felt a lack of information, and fear levels (1–10 point scale). Statistical analyses were performed at a significance level of *p* = 0.05.

**Results:**

The E group (313 respondents) proved to be better informed than WE (271). The lowest knowledge scores in both groups were related to pain medication and postoperative diet; the most unclear areas included complications (33.6%) and pain medication (22.1%). The highest level of effectiveness was observed for the combination of written leaflets and verbal instructions among the educational approaches evaluated. In E group, the mean fear score was 5.7 (SD = 2.7) before the first and 4.9 (SD = 2.8) before the second extraction (*p* < 0.001), while in WE the score was higher 6.3 (SD = 2.7) (*p* = 0.012). Fear levels were lower among regular dentist attendees (*p* = 0.004), and in men (*p* < 0.0001).

**Conclusion:**

Insufficient knowledge of postoperative care, especially in pain medication use, was observed, even among respondents with a prior history of the surgical extraction. Combined verbal and written instructions appeared to be associated with knowledge of preventive recommendations. Lower levels of fear were reported by men and by individuals with prior experience of the procedure.

## Introduction

The prevalence of eruption disturbances affecting the mandibular third molar reported in the literature may be as high as 73% [1–4]. Consequently, the surgical removal of impacted third molars remains among the most commonly performed procedures in dentoalveolar surgery. Despite being a routine procedure, it is associated with a relatively high incidence of postoperative morbidity, including bleeding, pronounced swelling, pain, alveolar osteitis, trismus, and other related complications [5, 6].

The risk of postoperative complications can be reduced by adhering to appropriate preventive recommendations. These include abstaining from nicotine-containing products, the adequate use of prescribed analgesics, eating a soft and non-irritating diet, and the maintenance of optimal oral hygiene both prior to and following the procedure [7–10]. The adjunctive use of antimicrobial mouth rinses after extraction may further contribute to complication prevention; however, their use should be initiated no earlier than the second postoperative day [11, 12]. Postoperative management may involve maintaining an elevated head position, applying cryotherapy to the surgical site, and implementing further supportive care strategies [13, 14]. Failure to follow these recommendations, along with the presence of dental plaque and gingivitis, increases the likelihood of post-extraction complications.

Public awareness and knowledge regarding the surgical extraction of third molars remains insufficiently documented in the literature. Limited understanding of the procedure and postoperative management in the population may contribute to heightened preoperative fear, consequently resulting in postponement or complete avoidance of the indicated procedure. Therefore, the aim of this study was to evaluate public knowledge regarding the procedure itself, postoperative management, preventive recommendations, and fear levels, both among individuals who had previously undergone surgical extraction of third molars (E) and those without such experience (WE). Furthermore, the effectiveness of the form of comprehensive information delivery was assessed among E respondents.

## Materials and methods

A questionnaire survey was conducted after acquiring local ethical committee approval EK-VP/56/00/2025. The questionnaire was designed for the general public, with a minimum age requirement of 18 years and no upper age limit, and was distributed electronically via Google Forms (Google LLC, Mountain View, USA) on social media platforms. The survey respected the guidelines of the Declaration of Helsinki, and informed consent was provided by the participants themselves while starting the questionnaire. The questionnaire was developed based on previously published literature [15], and further refined through expert consensus among three specialists in the field, incorporating additional items assessing fear levels and methods of information delivery related to the surgical procedure. It was subsequently pilot-tested on a sample of 25 participants to assess clarity and comprehensibility; no major modifications were required following the pilot phase. During the study period six months, 606 questionnaires were received; after excluding incomplete responses, the dataset comprised 584 respondents.

The questionnaire began with a set of demographic and background questions covering age, sex, general health status, smoking habits, frequency of visits to a dentist and dental hygienist, and the level of fear related to dental visits and the surgical third molar extraction. The fear level was assessed using a 10-point Anxiety Scale (AS). Scores on this 10-point scale can range from 1 (not anxious) to 10 (extremely anxious). Afterwards, participants reported whether they had previously undergone surgical extraction of third molars, being allocated to either E group or WE group.

This was followed by eight questions assessing knowledge of preventive recommendations before and after surgical extraction of third molars (with five response options comprising one correct answer categorized as “right”; three incorrect alternatives and an additional “I do not know” option, which were categorized as a „wrong“ response), awareness of the procedure itself (also by choosing from five response options), and areas in which respondents felt they lacked information (regarding anesthesia, pain medication, oral hygiene, dietary regimen, and postoperative complications). All participants in the E group responded to additional questions regarding their level of fear before a possible second procedure, employing the same 10-point AS. They were also asked how they had received information about the procedure and the associated preventive recommendations - verbally only by dentist or nurse; in writing and verbally; or not at all (obtaining the information themselves).

The data were entered into an Excel spreadsheet and analyzed statistically using IBM SPSS Statistics for Windows, Version 23.0 (Armonk, NY: IBM Corp.). All statistical analyses were performed at a significance level of *p* = 0.05. Quantitative variables were summarized using means, medians, and standard deviations (SD). The normality of data distribution was assessed using the Shapiro–Wilk test, which indicated that most variables were not normally distributed; therefore, non-parametric methods were applied. The Mann–Whitney U test was used to compare two independent groups (e.g., frequency of surgical extraction with increasing age in the sample) for quantitative and ordinal variables. When more than two independent groups were compared (e.g., attendance at regular dental check-ups in relation to fear of surgical extraction), the Kruskal–Wallis test was employed. For paired data (e.g., comparisons of responses before and after the procedure), the Wilcoxon signed-rank test was applied.

Associations between categorical variables were evaluated using the Chi-square test or Fisher’s exact test, as appropriate. All applied statistical tests inherently account for sample size when calculating p-values.

## Results

Among the 584 respondents, there were 112 (19.2%) men and 472 (80.8%) women; more than two-thirds were aged between 18 and 35 years (Table 1). All participants were Czech nationals. The majority of the respondents attended regular dental check-ups (88.7%). 313 (53.6%) respondents had personal experience with the surgical extraction of third molars.

**Table 1:** Demographic characteristics of the respondents, prevalence of nicotine product use, frequency of dental visits, anxiety before the visit, and prior history of surgical third molar extraction

Question (*n* = 584)		Number	%
Sex	Men	112	19.2%
	Women	472	80.8%
Which age group do you belong to?	18–25 years	213	36.5%
	26–35 years	192	32.9%
	36–50 years	139	23.8%
	over 50 years	40	6.8%
Do you smoke or consume nicotine in another form?	I smoke fewer than 10 cigarettes per day	44	7.5%
	I smoke more than 10 cigarettes per day	34	5.8%
	I do not smoke	468	80.1%
	I use other nicotine products	38	6.5%
Do you attend regular preventive check-ups with your dentist? (at least once a year)	Yes	518	88.7%
	No, only when in pain	35	6.0%
	I am not registered with a dentist	31	5.3%
Do you generally feel anxious before dental appointments?	Yes, before every visit	178	30.5%
	No, I do not feel anxious before dental appointment	256	43.8%
	Only before planned treatment	150	25.7%
Do you have personal experience with the surgical extraction of a third molar?	Yes. I have undergone this procedure	313	53.6%
	No	271	46.4%

A significant association between regular dental visits and more frequent experience with surgical extraction was proven (*p* = 0.016; Chi-square test), and the proportion of respondents who had undergone surgical extraction increased with age (*p* < 0.0001; Mann–Whitney U test). Most respondents were non-smokers (80.1%), and the highest proportion of nicotine users (10.0%) was found in the 18–24-year-old group (*p* = 0.024; Chi-square test).

Of all participants, 528 respondents correctly answered the questions on the need for radiographs before surgical extraction (E = 93.0%; WE = 87.5%; *p* = 0.034; Fisher’s exact test), and 504 on the use of local anesthesia (E = 92.7%; WE = 79.0%; *p* < 0.0001; Fisher’s exact test; Table 2). Most respondents also correctly answered that eating after extraction should be postponed until anesthesia has worn off (E = 80.5%; WE = 63.6%; *p* < 0.0001; Fisher’s exact test). The fewest correct answers were given for questions regarding eating (E = 39.0%; WE = 17.3%; *p* < 0.0001; Fisher’s exact test) and the use of pain medication before the procedure (E = 24.3%; WE = 15.1%; *p* = 0.007; Fisher’s exact test). Significant differences in knowledge were found between WE and E respondents; those with prior experience were generally better informed, except regarding smoking cessation after the procedure (Table 2). Regular dental attendance did not correlate with better knowledge of preventive recommendations related to surgical extraction, except for knowledge about eating before the procedure (*p* = 0.009; Fisher’s exact test).

**Table 2:** Correct and incorrect answers in the WE and E groups about pre- and postoperative care and preventive recommendations

Question/Answer		Do you have personal experience with surgical extraction of third molars?				*P*
		E		WE		
		Number	%	Number	%	
Can you eat before the procedure?	right	122	39.0%	47	17.3%	<0.0001***
	wrong	191	61.0%	224	82.7%	
Can you take painkillers before the procedure?	right	76	24.3%	41	15.1%	0.007**
	wrong	237	75.7%	230	84.9%	
Is anesthesia used during the procedure?	right	290	92.7%	214	79.0%	<0.0001***
	wrong	23	7.3%	57	21.0%	
Is it necessary to take an X-ray before the procedure?	right	291	93.0%	237	87.5%	0.034*
	wrong	22	7.0%	34	12.5%	
Can you eat immediately after the procedure or when the anesthesia wears off?	right	252	80.5%	172	63.5%	<0.0001***
	wrong	61	19.5%	99	36.5%	
Is it advisable to rinse your mouth immediately after the procedure?	right	127	40.6%	74	27.3%	0.0009***
	wrong	186	59.4%	197	72.7%	
Should you avoid smoking after the procedure?	right	136	43.5%	118	43.5%	1.000
	wrong	177	56.5%	153	56.5%	
Can you take painkillers after the procedure?	right	147	47.0%	66	24.4%	<0.0001***
	wrong	166	53.0%	205	75.6%	

*p < 0.05; *p < 0.01; **p < 0.001. E —third molar surgical extraction group; WE — non-extraction group

**Fig. 1:**
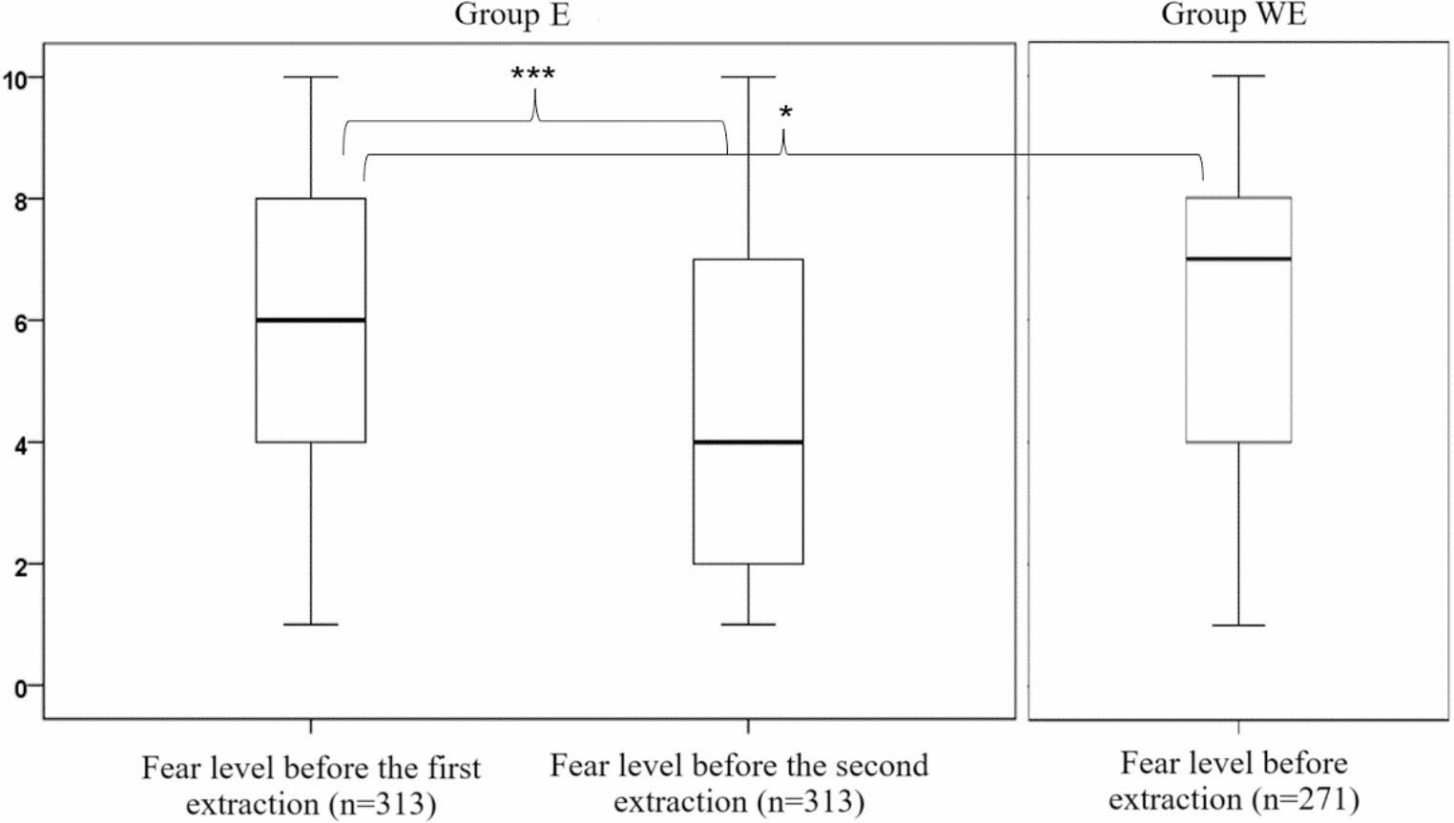
Fear levels before the first and second third molar surgical extractions in E group and before the first extraction in WE group (*p<0.05; ***p<0.001).

Among E respondents, the mean fear level before surgical extraction of third molars was rated at 5.7 (SD = 2.7) before the first extraction, and dropped to 4.9 (SD = 2.8) before the second extraction (*p* < 0.001; Wilcoxon signed–rank test; Fig. 1). In the WE group, the fear level was rated at 6.3 (SD = 2.7), being significantly higher than in the E respondents before their first procedure (*p* = 0.012; Mann–Whitney U test).

Respondents who attended regular dental check-ups reported lower fear of surgical extraction (*p* = 0.004; Kruskal–Wallis test; Table 3). In both groups of respondents, anxiety before a dental visit was reported by 178 (30.5%) of them prior to every appointment, by 150 (25.7%) only before planned treatment, and was absent in 256 (43.8%) of all respondents. Those who were anxious before dental visits in general reported greater fear of surgical extraction (*p* < 0.0001; Mann–Whitney U test). Men reported significantly lower fear than women before the surgical extraction of the third molar (*p* < 0.0001; Mann–Whitney U test), both before the first and second procedures. A weak negative correlation was found between age and fear before the procedure (*r* = − 0.2; *p* < 0.0001; Spearman’s rank correlation).

**Table 3:** Comparison of correct answers in the E group based on the form of receiving information about the procedure and of pre- and postoperative care

Question/answer		In what form were you informed about postoperative care?						*p*
		I was not informed; I obtained the information myself		In writing and verbally		Verbally only, by the dentist/nurse		
		Number	%	Number	%	Number	%	
Can you eat and drink before the procedure?	right	6	42.9%	64	47.1%	52	32.1%	0.027*
	wrong	8	57.1%	72	52.9%	110	67.9%	
Can you take painkillers before the procedure?	right	2	14.3%	35	25.7%	39	24.1%	0.704
	wrong	12	85.7%	101	74.3%	123	75.9%	
Is anesthesia used during the procedure?	right	9	64.3%	128	94.1%	152	93.8%	0.004*
	wrong	5	35.7%	8	5.9%	10	6.2%	
Is it necessary to take an X-ray before the procedure?	right	11	78.6%	130	95.6%	149	92.0%	0.044*
	wrong	3	21.4%	6	4.4%	13	8.0%	
Can you eat immediately after the procedure?	right	12	85.7%	113	83.1%	127	78.4%	0.602
	wrong	2	14.3%	23	16.9%	35	21.6%	
Is it advisable to rinse your mouth immediately after the procedure?	right	3	21.4%	66	48.5%	58	35.8%	0.029*
	wrong	11	78.6%	70	51.5%	104	64.2%	
Should you avoid smoking after the procedure?	right	3	21.4%	61	44.9%	72	44.4%	0.251
	wrong	11	78.6%	75	55.1%	90	55.6%	
Can you take painkillers after the procedure?	right	4	28.6%	69	50.7%	74	45.7%	0.253
	wrong	10	71.4%	67	49.3%	88	54.3%	
Clarity of received information (regarding anesthesia, pain medication, oral hygiene, dietary regimen, and postoperative complications)	1 to 3 areas unclear	13	92.9%	119	88.1%	148	91.9%	0.660
	4 or more areas unclear	1	7.1%	7	5.2%	7	4.3%	
	Everything was clear	0	0.0%	9	6.7%	6	3.7%	

*p < 0.05

Within the E group, respondents were categorized according to how they received information: verbally, verbally combined with a written leaflet, or not at all (i.e., relying on self-sourced information) (Fisher’s exact test; Table 3). Overall, a consistent pattern emerged: respondents who received both verbal and written information achieved the highest proportion of correct answers, while those informed only verbally or not at all performed worse.

Specifically, for the question “Can I eat before the procedure?”, the highest proportion of correct responses was observed among respondents informed both verbally and in writing (47%), compared to 32% among those informed only verbally (*p* = 0.027). A similar trend was observed for rinsing after extraction, where combined verbal and written information was associated with higher correctness (49%; *p* = 0.029). In contrast, respondents who were not informed at all showed significantly lower correct response rates for the question “Is anesthesia used during the procedure?” (64% correct; *p* = 0.004) and for the necessity of a radiograph (79% correct; *p* = 0.044).

When asked about perceived lack of information regarding the surgical extraction of the third molar, respondents could select multiple options. Among E respondents, the most commonly unclear areas were complications (33.6%) and use of pain medication (22.1%), followed by eating before (16.2%) and anesthesia during the procedure (14.2%). Oral hygiene rules after the procedure were unclear to 10.1% of respondents. Those with four or more unclear areas were classified as “uninformed,” and those with one to three unclear areas were “insufficiently informed” (Table 4). WE respondents more often had four or more unclear areas (9.3%) compared to E respondents (4.8%), while E respondents more often reported that everything was clear (4.8% vs. 1.9%) (*p* = 0.018; Fisher’s exact test).

**Table 4:** Number of unclear areas in which respondents from E and WE groups felt a lack of information

Clarity of information		E	WE	Total
1 to 3 areas unclear	Number	281	240	521
	%	89.8%	88.6%	89.2%
4 or more areas unclear	Number	15	25	40
	%	4.8%	9.2%	6.8%
Everything was clear	Number	15	5	20
	%	4.8%	1.8%	3.4%
Other	Number	2	1	3
	%	0.6%	0.4%	0.5%
Total	Number	313	271	584
	%	100.0%	100.0%	100.0%

E —third molar surgical extraction group; WE — non-extraction group

## Discussion

The results suggest that the questionnaire distributed via social media was predominantly completed by individuals with an interest in both general and dental health — most attended regular dental check-ups and did not smoke or use other nicotine products. Paradoxically, the highest proportion of smokers and users of nicotine products was observed in the youngest age group (18–24 years), which is consistent with the findings of the National Survey on Tobacco and Alcohol Use in the Czech Republic for 2023 [16]. Additionally, a pronounced predominance of female respondents was observed, which may be associated with the generally greater interest in oral health among women reported in the literature [17]. Approximately, half of the respondents had undergone third molar extraction, mainly among those attending regular dental check-ups, as might be anticipated.

Fear is an unpleasant emotion that arises as a response to a real threat or danger—it is an emotional reaction to perceived harm. The average level of fear reported by respondents before surgical extraction was relatively high, rated at approximately 6 on a scale from 1 to 10. Literature confirms that invasive dental procedures, in particular, tend to cause fear in patients [18, 19]. In those respondents who had already undergone surgical extraction of third molars, fear levels before the second extraction significantly decreased, indicating that fear of the “unknown” contributes to preoperative anxiety.

Those who reported fear of visiting the dentist in general were also more fearful of the extraction itself, which aligns with findings in the literature [20]. Interestingly, those who underwent the surgical extraction were less fearful even before their first extraction, suggesting that fear itself may be a factor contributing to the postponement of indicated surgical extraction, a relationship also documented for other dental procedures [20, 21]. Furthermore, men reported lower levels of anxiety than women prior to both the first and second procedures, which is consistent with findings from previous studies on dental anxiety [17, 22–24]. However, this result should be interpreted with caution, as women in the present study were significantly overrepresented among the respondents compared with men. A weak but significant correlation between increasing age and decreasing fear was also found, possibly linked to the higher proportion of older respondents who had already undergone other dental procedures, thus reducing the fear. Increased age is also noted in the literature as a factor that lowers dental anxiety [22, 25, 26].

As expected, the knowledge level of E respondents regarding the procedure itself and preventive recommendations was higher than that of WE respondents. Those who had undergone the procedure were generally better informed, indicating adequate pre- and postoperative patient education in most cases. In contrast, no difference was observed between E and WE respondents in knowledge of post-extraction smoking restrictions, which may be explained by the overall low prevalence of smokers in our sample. Furthermore, the respondents in the E group probably reported being non-smokers in their medical history, hence information on the adverse effects of smoking on oral wound healing may not have been routinely provided prior to surgical extraction. Nevertheless, the results suggest that public awareness of smoking-related complications after extraction appears to remain insufficient, consistent with findings reported by Larrazábal et al. in 2010 [9].

For both WE and E groups, the lowest proportion of correct answers was found for questions on eating before the procedure and the use of pain medication before and after extraction. This is notable, as paracetamol and ibuprofen have been proven effective in managing postoperative pain and reducing complications, although one review suggests otherwise [7, 10, 27]. The issue of insufficient knowledge on the use of over-the-counter pain medication in relation to the surgical procedure was evident in both groups of respondents and is in accordance with the literature findings [15]. These results indicate that patient education in the areas of pain management following surgical extraction should be more widely disseminated.

Among respondents who regularly attended preventive dental check-ups, only knowledge about the possibility of eating before the procedure was better compared to those who did not; other areas of awareness were equally uncertain. This suggests that regular attendance at the dentist alone does not improve knowledge about surgical extraction, as education is typically provided directly by the dental care team or oral surgeon before or after the procedure.

Other potential preventive recommendations that were not investigated by the present research include head elevation and external cryotherapy after the surgical extraction. However, “head elevation” is a relative concept that may be interpreted differently by patients and is therefore not regarded as a standardized intervention [7]. Conversely, applying cryotherapy to the surgical site is generally recommended to prevent complications after extraction, but there is no consensus regarding the exact method and timing after surgical extraction of impacted third molars [7, 13, 28]. Incorrect application can itself cause complications, and its benefits remain debated [14].

The present study also evaluated the method of education regarding preventive recommendations. Respondents, who received combined written and verbal instructions, demonstrated a higher level of knowledge. Solely verbal recommendations proved less informative, likely because stress before and immediately after surgery might make it difficult for patients to remember the instructions properly.

When asked to identify areas where they lacked sufficient information, one-third of E respondents indicated “complications” and over one-fifth “use of pain medication” as unclear, followed by “diet” and “anesthesia during the procedure.” About 10% were uncertain about oral hygiene rules. These results are suboptimal, given that by law every patient must be informed about all risks of the procedure in clear terms they can understand [29]. Such information should also be summarized in the written informed consent (IC) signed before surgery. The IC typically lists the general risks associated with the procedure, but these should always be supplemented with risks relevant to the individual patient’s age, medical history, and comorbidities. On the other hand, overloading patients with all possible risks could cause unnecessary stress, as patients tend to overestimate the likelihood of postoperative complications [30]. However, risks that could influence patient decision-making must not be withheld. Direct personal communication, supplementing the IC and an informational leaflet, together with the opportunity to ask questions before the procedure, seems appropriate.

Limitations of the study: This study is subject to several potential sources of bias. Participants were not selected through random sampling, which may limit the representativeness of the sample and reduce the generalizability of the findings to the broader population. The questionnaire was likely completed predominantly by individuals with heightened interest in oral health and greater health awareness, which may have contributed to an overestimation of correct responses. Furthermore, the distribution of the questionnaire via social media limited the study sample to those with regular access to the internet. The marked predominance of female respondents should also be taken into consideration. In addition, the data relied on self-reported information, which may be affected by recall bias, particularly when participants were asked to report over a longer time interval. Consequently, some responses may be imprecise.

## Conclusion

Insufficient knowledge of postoperative care, especially in pain medication use, was observed, even among respondents with a prior history of the extraction. Respondents who received both verbal and written recommendations demonstrated better knowledge, indicating that this combined approach may represent an effective method of patient education. In the future, patient education should focus more on the proper use of pain medication, postoperative dietary recommendations, and possible complications. Most respondents feared the procedure; men, older respondents, and those with prior experience of the procedure reported lower fear levels.

## Data Availability

The datasets analysed during the current study are available from the corresponding author upon reasonable request.

## Declarations

### AI usage

AI-assisting was used only to improve the readability and language of the article with human oversight and control.
